# A risk scoring tool for predicting Kenyan women at high risk of contraceptive discontinuation

**DOI:** 10.1016/j.conx.2020.100045

**Published:** 2020-10-29

**Authors:** Claire W. Rothschild, Barbra A. Richardson, Brandon L. Guthrie, Peninah Kithao, Tom Omurwa, James Mukabi, Erica M Lokken, Grace John-Stewart, Jennifer A. Unger, John Kinuthia, Alison L. Drake

**Affiliations:** a Department of Epidemiology, University of Washington, Seattle, USA; b Departments of Biostatistics and Global Health, University of Washington, Seattle, USA; Division of Vaccine and Infectious Diseases, Fred Hutchinson Cancer Research Center, Seattle, USA; c Departments of Epidemiology and Global Health, University of Washington, Seattle, USA; d University of Washington-Kenya, Nairobi, Kenya; e PATH-Kenya, Nairobi, Kenya; f Department of Global Health, University of Washington, Seattle, USA; g Departments of Global Health, Epidemiology, Medicine, and Pediatrics, University of Washington, Seattle, USA; h Department of Obstetrics and Gynecology, University of Washington, Seattle, USA; i Department of Research and Programs, Kenyatta National Hospital, Nairobi, Kenya

**Keywords:** Contraception, Family planning, Discontinuation, Unmet need, Risk score, LMIC

## Abstract

**Objective:**

We developed and validated a pragmatic risk assessment tool for identifying contraceptive discontinuation among Kenyan women who do not desire pregnancy.

**Study design:**

Within a prospective cohort of contraceptive users, participants were randomly allocated to derivation (*n* = 558) and validation (*n* = 186) cohorts. Risk scores were developed by selecting the Cox proportional hazards model with the minimum Akaike information criterion. Predictive performance was evaluated using time-dependent receiver operating characteristic curves and area under the curve (AUC).

**Results:**

The overall contraceptive discontinuation rate was 36.9 per 100 woman-years (95% confidence interval [CI] 30.3–44.9). The predictors of discontinuation selected for the risk score included use of a short-term method or copper intrauterine device (vs. injectable or implant), method continuation or switch (vs. initiation), < 9 years of completed education, not having a child aged < 6 months, and having no spouse or a spouse supportive of family planning (vs. having a spouse who has unsupportive or uncertain attitudes towards family planning). AUC at 24 weeks was 0.76 (95% CI 0.64–0.87) with 70.0% sensitivity and 78.6% specificity at the optimal cut point in the derivation cohort. Discontinuation was 3.8-fold higher among high- vs. low-risk women (95% CI 2.33–6.30). AUC was 0.68 (95% CI 0.47–0.90) in the validation cohort. A simplified score comprising routinely collected variables demonstrated similar performance (derivation-AUC: 0.73 [95% CI 0.60–0.85]; validation-AUC: 0.73 [95% CI 0.51–0.94]). Positive predictive value in the derivation cohort was 31.4% for the full and 28.1% for the simplified score.

**Conclusions:**

The risk scores demonstrated moderate predictive ability but identified large proportions of women as high risk. Future research is needed to improve sensitivity and specificity of a clinical tool to identify women at high risk for experiencing method-related challenges.

**Implications:**

Contraceptive discontinuation is a major driver of unmet contraceptive need globally. Few tools exist for identifying women who may benefit most from additional support in order to meet their contraceptive needs and preferences. This study developed and assessed the validity of a provider-focused risk prediction tool for contraceptive discontinuation among Kenyan women using modern contraception. High rates of early discontinuation observed in this study emphasize the necessity of investing in efforts to develop new contraceptive technologies and stronger delivery systems to better align with women's needs and preferences for voluntary family planning.

## 1 Introduction

Globally, an estimated 220 million women at risk for an unplanned pregnancy are not using contraception [[1], [2], [3]]. Meeting women's contraceptive needs is a priority for reproductive justice [4] and for improving maternal and newborn health [5,6]. Increasingly, discontinuation is recognized as a key driver of unmet contraceptive need [[7], [8], [9], [10]]. By 1 year, over one third of women using modern, reversible contraceptive methods discontinue contraception in low- and middle-income countries [11]. Method-related problems, such as side effects and difficulty using methods, are the most common reasons given for discontinuation [11,12]. Helping women who wish to avoid pregnancy but who experience contraceptive method-related challenges achieve their reproductive goals is critical for preventing unintended and mistimed pregnancy.

Current family planning (FP) guidelines emphasize tailoring counseling to individual needs and focusing on essential information, with the goal of helping women identify the contraceptive method that will best meet their personal needs [13]. Recent studies suggest that quality of FP counseling, including provision of method information and counseling on options for switching methods if dissatisfied, is protective against early discontinuation [[14], [15], [16], [17], [18]]. Several randomized trials have also found that low-intensity interventions such as SMS reminders result in improved continuation rates [[19], [20], [21]]. However, current counseling guidelines do not aid providers in identifying women at highest risk of method-related discontinuation who could benefit from FP counseling or support specifically focused on meeting contraceptive needs and preferences. Intensive counseling may be burdensome or irrelevant to many women seeking FP services. Given the reality of time-constrained FP visits, tools that can support providers to more effectively tailor their counseling messages are needed.

Risk scoring systems have been developed for a variety of adverse health outcomes in sub-Saharan Africa [[22], [23], [24]]. However, tools to identify women who may benefit most from additional counseling and support to ensure their contraceptive needs and preferences are met have not been constructed. We developed an empiric prediction tool that could be used by FP providers to identify women at highest risk for contraceptive discontinuation.

## 2 Methods

### 2.1 Study population

We used data from the Mobile Data Collection for Contraceptive Use, Behaviors and Experience (mCUBE) study, a prospective cohort study of women's contraceptive experiences. Study participants were enrolled February–May 2018 while attending FP or maternal and child health clinics within 10 public health facilities in 5 counties of Western Kenya (Bungoma, Homa Bay, Kakamega, Kisumu and Nyamira). Women were eligible if they were ≥ 18 years old (or an emancipated minor ≥ 14 years old with a previous pregnancy); had daily access to a mobile phone with a Safaricom SIM card; were able to read and respond to SMS in English, Swahili or one of two local languages (Luo or Kisii) either alone or with the help of a trusted person; and were currently initiating, continuing or switching to a modern, reversible contraceptive method. Modern methods included injectables, implants, intrauterine devices or systems (IUDs), oral contraceptive pills (OCPs), emergency contraceptive pills, condoms, diaphragms, lactational amenorrhea, Standard Days Method and TwoDay Method [25].

### 2.2 Data collection

Data were collected through structured SMS surveys operated by the Kenya-based company mSurvey (Nairobi, Kenya). Study staff administered an enrollment SMS survey, capturing information on sociodemographic characteristics, reproductive and contraceptive history, contraceptive use, fertility goals, and perceived quality and satisfaction with FP services. Participants received weekly follow-up SMS surveys for 24 weeks that captured information on contraceptive use, method type, reasons for switch or discontinuation (if applicable), side effects and healthcare utilization. Details on contraceptive method and discontinuation ascertainment are provided in the Online Appendix.

### 2.3 Ethical considerations

All study procedures were approved by the Maseno University Ethical Review Committee. Participants signed a written consent form prior to any study procedures. The University of Washington's (UW's) Human Subjects Division (HSD) determined that ethical approval from UW was not required as the UW research team was not considered engaged in human subjects research; however, this specific analysis was approved by UW HSD.

### 2.4 Risk score development and validation

Contraceptive discontinuation was defined as a period of ≥ 2 consecutive weeks during which women self-reported that they were not currently using any modern contraceptive method. We defined discontinuation based on a ≥ 2-week period in order to capture short-term discontinuation episodes that have not been widely explored in the published literature. Method switches were considered as continuation unless a ≥ 2-week period elapsed with no modern method use. Our analytic sample comprised participants with complete baseline data for all risk factors considered and at least one complete observation during follow-up. Women who desired a pregnancy in the next year were excluded, as they were expected to be more likely to discontinue to become pregnant rather than for method-related reasons [26]. Potential predictors considered for the risk score included sociodemographic and clinical characteristics routinely collected in Kenyan FP clinics. Additional potential predictors not routinely collected (education, whether her spouse supported her contraceptive use, feelings about a hypothetical near-term pregnancy, side effects history and perceived quality of FP care) were also evaluated.

For score development and validation, 75% were randomly assigned to a derivation cohort to select the prediction model and the remaining 25% to a validation cohort. Due to the relatively high level of interval censoring (3194/15,266 or 21% of weekly observations), we imputed weekly self-reported method use by carrying forward the last observation and carrying backward the next observation; we did not impute after a participant's final complete weekly report (Online Appendix). In the derivation sample, stepwise selection was used to identify the Cox proportional hazards model with the minimum Akaike information criterion [27]. If potential predictors were collinear in the full sample, the variable with a greater scientific basis for inclusion based on the published literature was included prior to model selection. Covariates considered in model selection are in Table 1. A full risk score model using all variables selected in the stepwise model as well as a simplified model comprising variables routinely collected in FP clinics was created.

**Table 1:** Sociodemographic, reproductive and FP characteristics of the derivation and validation cohorts at study enrollment

	Derivation cohort (*n* = 558)	Validation cohort (*n* = 186)	
	*n* (%)	*n* (%)	p value
**Sociodemographic characteristics**			
Age category (years)			
≤ 20	43 (8)	16 (9)	.66
21–25	211 (38)	77 (41)	
26–30	153 (27)	53 (28)	
31–35	87 (16)	24 (13)	
> 35	64 (11)	16 (9)	
Completed education < 9 years	283 (51)	93 (50)	.87

*Relationship status*			
Not married (legal or presumed)	103 (18)	36 (19)	.96
Spouse supportive of FP	422 (76)	139 (75)	
Spouse not supportive of FP or unsure of spousal support	33 (6)	11 (6)	

**Reproductive characteristics**			
Number of living children [median (IQR)]	2 (1–3)	2 (1–3)	.43
Does not have a child aged < 6 months	392 (70)	132 (71)	.85

*Fertility intentions*			
Unsure intention to have children or unsure of preferred timing	116 (21)	31 (17)	.55
Desires no future children	121 (22)	38 (20)	
Desires next pregnancy in 1–2 years	48 (9)	19 (10)	
Desires next pregnancy in > 2 years	273 (49)	98 (53)	

*Pregnancy in the short-term future would be:*			
Not sure	72 (13)	27 (15)	.77
A big problem	315 (56)	98 (53)	
A small problem	48 (9)	15 (8)	
No problem	123 (22)	46 (25)	

**Characteristics of FP services received**			
Contraceptive method type			
Injectables	226 (41)	84 (45)	.50
Implant	241 (43)	79 (42)	
Intrauterine device (Cu-IUD/IUS)	376 (6)	10 (5)	
Pills^a^	31 (6)	5 (3)	
Other modern^b^	24 (4)	8 (4)	

FP user type			
Initiating contraception	146 (26)	48 (26)	.89
Switching from one method type to another	70 (13)	21 (11)	
Continuing method used in past month	342 (61)	117 (63)	

History of contraceptive side effects			
No	289 (52)	95 (51)	.14
Yes	258 (46)	91 (49)	
Unsure	11 (2)	0 (0)	
Traveled less than 30 min to reach health facility	351 (63)	118 (63)	.90

**Quality of care and satisfaction**			
“Very satisfied” with services received	267 (48)	86 (46)	.70
Felt that her privacy was not protected during the visit	33 (6)	14 (8)	.43
Felt provider gave:			
Accurate information	50 (9)	15 (8)	.71
Inaccurate information or unsure of accuracy	508 (91)	171 (92)	
Felt provider's treatment was “very respectful”	493 (88)	160 (86)	.40
Number of items in Method Information Index received [median (IQR)]^c^	3 (1–3)	3 (1–3)	.81

Feelings about using FP:			
No fears or concerns	353 (63)	127 (68)	.35
Reported having fears or concerns	184 (33)	55 (30)	
Unsure of having fears or concerns	21 (4)	4 (2)	

Notes: p values obtained using *χ*^2^ test for proportion or Wilcoxon rank-sum test of medians for continuous measures.

a Pills include daily combined and progestin-only oral contraceptives.

b Other modern methods include condoms, fertility-awareness-based methods (LAM, TwoDay Method, Standard Days Method) and emergency contraceptive pills.

c The Method Information Index is calculated based on three questions: “During your visit, (1) were you informed about other methods? (2) Were you informed about side effects or problems with the method? (3) Were you told what to do if you had side effects of problems with the method?”. Responses were summed to provide a count of the number of counseling items received, from 0 (received none of these counseling items) to 3 (received all items).

To construct risk scores, points were assigned to each variable by taking the ratio of its coefficient to the minimum coefficient in the multivariable Cox model rounded to the nearest integer [22,23]. We assessed predictive value of the risk score using receiver operating characteristic (ROC) curves and area under the curve (AUC) estimates at 12 and 24 weeks using an inverse-probability-of-censoring-weighting approach for right-censored data (Online Appendix) [28]. The 12- and 24-week time points were selected to assess rapid discontinuation after uptake and at the maximum follow-up time, respectively. Time-dependent sensitivity, specificity, positive predictive value (PPV) and negative predictive value (NPV) were estimated, with optimal cut points defined by Youden's *J* statistic. Risk score performance was evaluated in the validation and full cohorts using time-dependent AUC-ROC analysis.

Several sensitivity analyses were conducted. First, in addition to excluding women desiring pregnancy in the next year, the primary risk score was reconstructed additionally excluding women who desired a future pregnancy but were unsure when. Second, the full risk score was fit using a subdistribution hazard model [29], with discontinuation for pregnancy desire as a competing risk. We modeled missing reason for discontinuation using multiple imputation with chained equations [30]. Third, we explored an alternative methodology for variable selection using the Cox extension of the least absolute shrinkage and selection operator (LASSO) with a grouped penalty for categorical covariates (Online Appendix). Comparative performance of the stepwise- and LASSO-Cox risk scores is of interest, as LASSO may reduce overfitting compared to standard stepwise approaches [31]. Finally, we rederived the risk score using an alternative definition of discontinuation that required 4, rather than 2, weeks of method nonuse to assess robustness of our findings to selection of the discontinuation interval. Additional sensitivity analyses to assess the potential impact of measurement error in self-reported method use are presented in the Online Appendix. All analyses were conducted in Stata v15.1 (StataCorp, College Station, TX, USA) and R v3.6.2 (The R Project for Statistical Computing).

## 3 Results

**Fig. 1:**
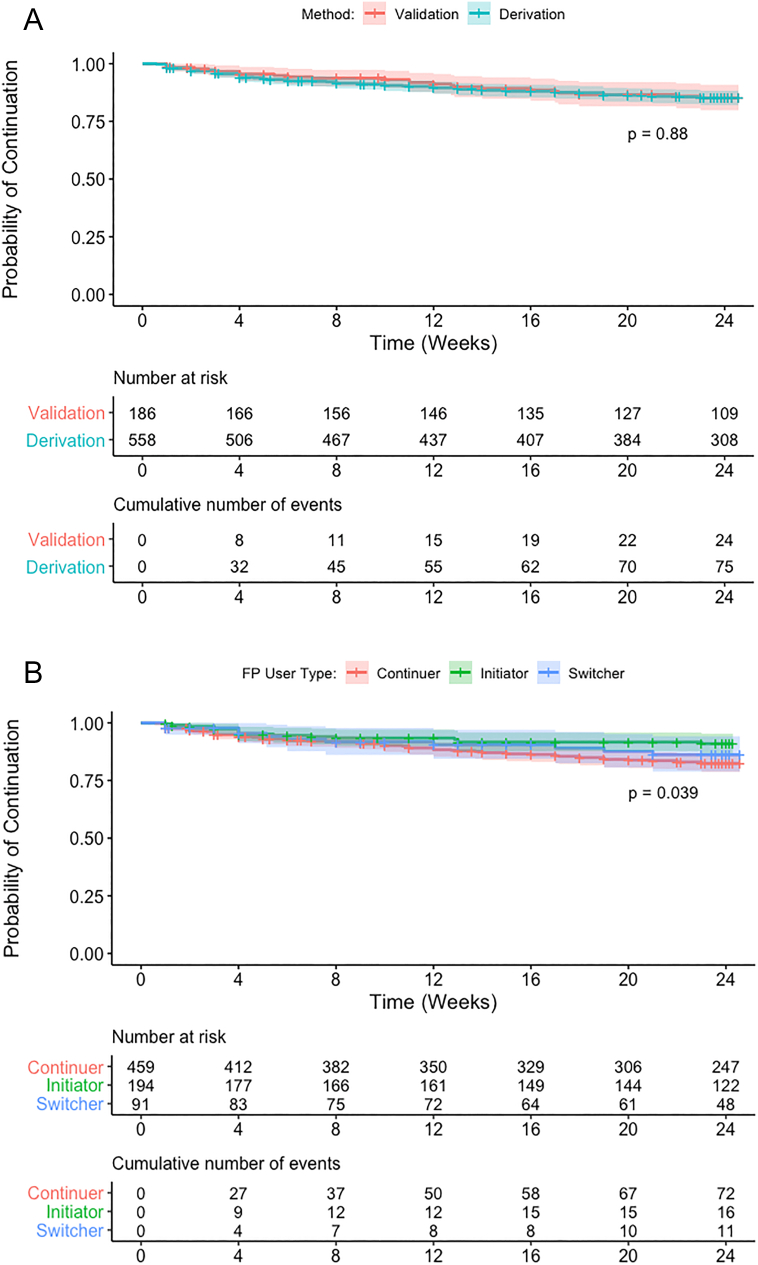
(A) Contraceptive discontinuation by 24 weeks, by cohort. Notes: p values calculated using the regular log-rank test with weights equal to 1. (B). Contraceptive discontinuation by 24 weeks, by FP user type (method initiator, switcher or continuer). Notes: p values calculated using the regular log-rank test with weights equal to 1.

Of 1212 mCUBE study participants, 744 (61%) were included in this analysis; 255 (21%) were excluded due to missing enrollment characteristics, 91 (8%) due to missing follow-up, 68 (6%) due to missing both enrollment and follow-up data and 54 (4%) due to reported desire to become pregnant within 1 year. Among participants with complete enrollment data, we observed no differences in having any completed follow-up (defined as response to at least one weekly survey) or duration of completed follow-up by method type. Additional details on characteristics of the analytic sample are provided in the Online Appendix. Median age was 26 years (interquartile range [IQR] 23–31), and half (51%) reported completing < 9 years of education (Table 1). Most (94%) women had at least one prior pregnancy, with 30% having a child < 6 months old. Three quarters of participants reported having a partner who supported her FP use. Implants (43%) and injectables (42%) were the most prevalent contraceptive methods at enrollment. The full cohort contributed 268.2 woman-years of follow-up with 99 incident contraceptive discontinuation events, for an overall incidence of 36.9 per 100 woman-years (95% confidence interval [CI] 30.3–44.9) (Fig. 1). Overall, 38% of women were initiating contraception or switching to a new contraceptive method at enrollment. Among these women, we observed a discontinuation rate of 25.7 per 100 woman-years (95% CI 17.6–37.5 per 100 woman-years), corresponding to a 12-month cumulative incidence of 22.7% (Fig. 1). Discontinuation rates by user and method type are provided in the Online Appendix. Most (119/194, 61%) women newly initiating contraception were motivated by a change in their perceived risk of unintended pregnancy, while 15% (30/194) cited birth spacing and 9% (17/194) a recent increase in sexual activity. Among method switchers, 60% (55/91) switched due to side effects or health concerns with their prior method. There were few differences between the derivation and validation cohorts. Compared to participants included in the analysis, excluded participants had lower education attainment and were more likely to desire no future children, use other modern methods (condoms or fertility-awareness methods) and report fears about using FP (Online Appendix).

### 3.1 Risk score development

**Fig. 2:**
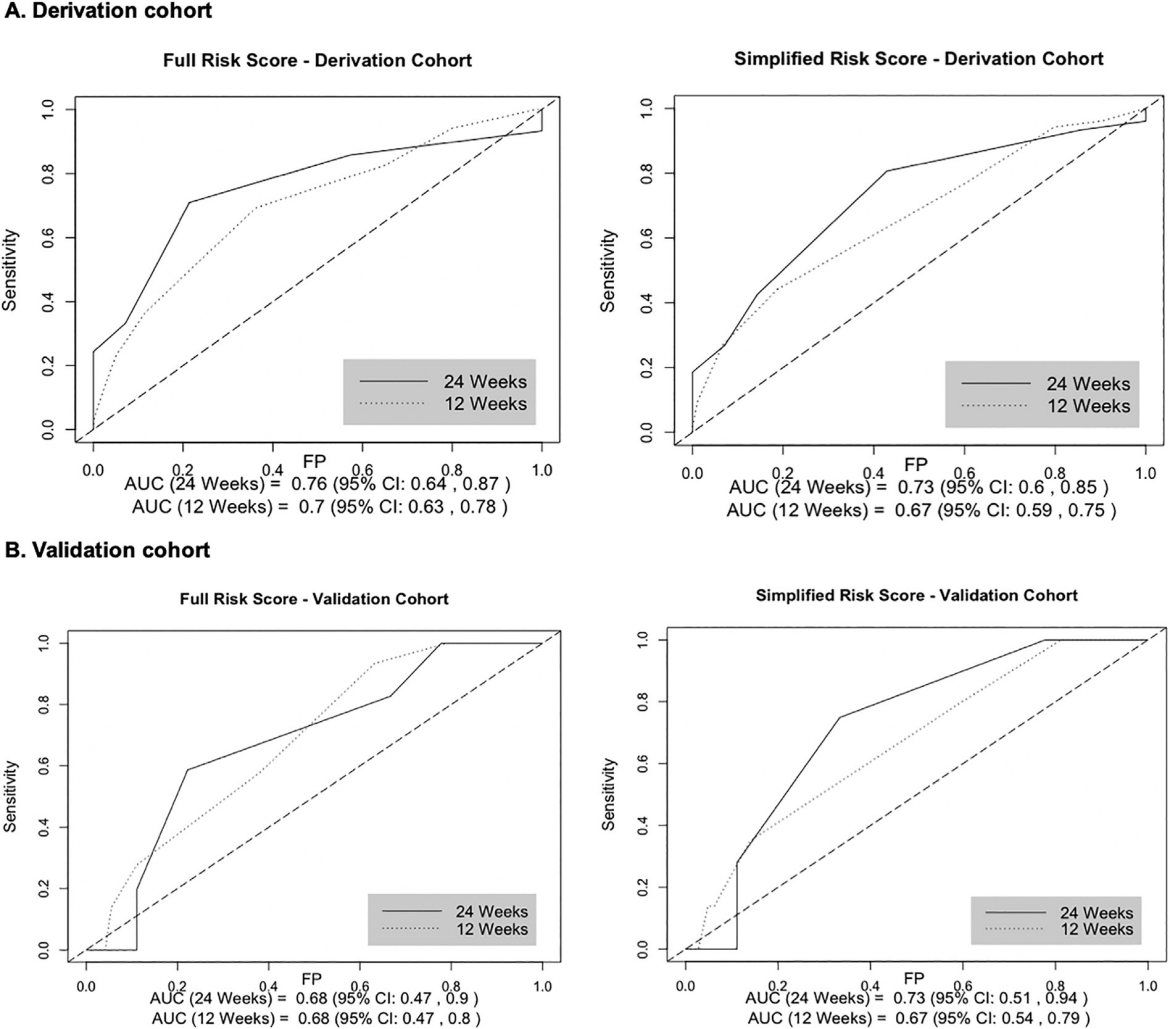
Receiver operating characteristic curve and optimal cut points of risk scores.

**Fig. 3:**
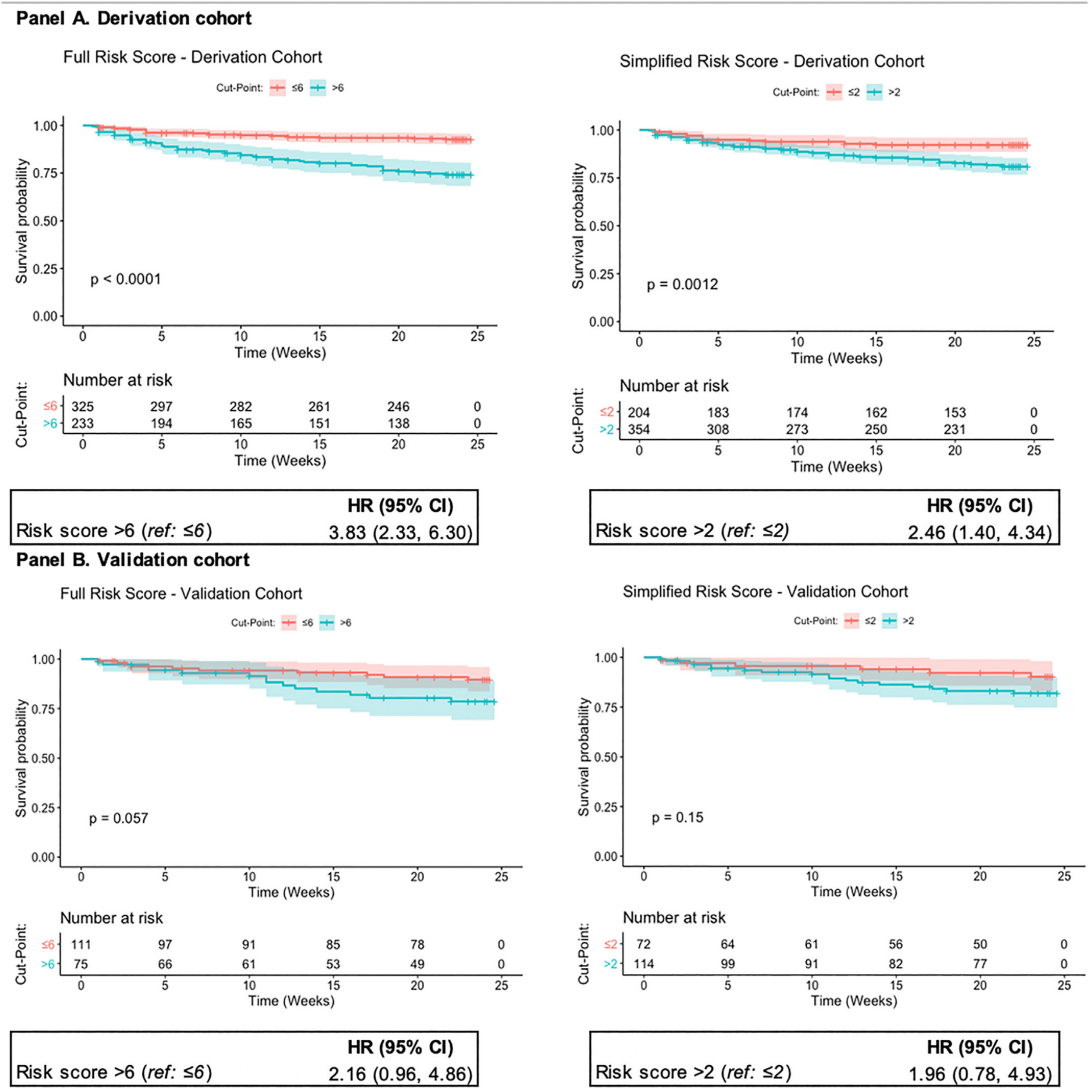
Survival probabilities by optimal cut points of risk scores. Notes: p values calculated using the regular log-rank test with weights equal to 1.

Incidence of discontinuation in the derivation cohort was 37.3 per 100 woman-years (95% CI 29.7–46.7) (Fig. 1). The full risk score included the following variables: method type, continuing or switching methods, vs. contraceptive initiation, < 9 years of completed education, not having a child aged < 6 months, being unmarried and having a spouse supportive of FP (Table 2). Injectables and implants were combined into a single reference category for method type; this post hoc decision was based on the similar risk of discontinuation observed among women using these methods (adjusted hazard ratio: < 1.01). Due to the small number of women using emergency contraceptive pills, fertility-based methods and condoms in our sample, these methods were also grouped in a single category in the analysis. Reporting an unsupportive spouse or being uncertain of spouse's support for FP was associated with contraceptive continuation. The median score in the derivation cohort was 6 (IQR 5–7; range 0–12) (Table 2). The AUC was 0.76 at 24 weeks (95% CI 0.64–0.87) and 0.70 at 12 weeks (95% CI 0.63, 0.78) (Fig. 2A). At the optimal cut point of 6, 41.8% (*n* = 233) were identified as high risk, with 70.9% sensitivity, 78.6% specificity and 36.6% PPV at 24 weeks (Table 3). Discontinuation risk was 3.8-fold higher among women with risk scores > 6 vs. ≤ 6 (95% CI 2.33–6.30) (Fig. 3A).

**Table 2:** Adjusted hazard ratios of multivariable risk score models on contraceptive discontinuation in the derivation cohort.

	Full risk score		Simplified risk score	
	HR (95% CI)	Points	HR (95% CI)	Points
***Contraceptive method type***				
Cu-IUD/IUS	1.58 (0.67–3.72)	1	1.56 (0.66–3.66)	1
Pills^a^	3.16 (1.59–6.26)	3	3.32 (1.68–6.57)	3
Other modern^b^	5.32 (2.46–11.42)	4	4.95 (2.32–10.59)	4
*Reference: injectables, implants*	*Ref.*	0	*Ref.*	0

***FP user type***				
Continuing method used in past month	2.25 (1.14–4.23)	2	2.41 (1.23–4.73)	2
Switching from one method type to another	2.10 (0.88–5.06)	2	2.07 (0.87–4.97)	2
*Reference: initiating contraception*	*Ref.*	0	*Ref.*	0
**< 9 years of completed education**	1.62 (1.01–2.60)	1	--	--
**Does not have child aged <** **6 months**	1.60 (0.89–2.86)	1	1.57 (0.88–2.82)	1

***Relationship status***				
Spouse supportive of FP	4.58 (0.63–33.24)	3	--	--
Not married (legal or presumed)	7.34 (0.97–55.21)	4	1.70 (0.98–2.93)	1
*Reference: spouse unsupportive/unsure of spousal support*	*Ref.*	0	--	--
**Maximum score**		**12**		**8**

Notes: Variables included in the full risk score model were selected using stepwise forwards and backwards selection to identify the Cox model with the minimum AIC value. The Efron approach was used to handle ties. The simplified risk score was developed by removing features in the full risk score that are not routinely collected (either verbally or in written documentation) in Kenyan public health facilities. *β* is the Cox proportional hazards model coefficients (nonexpontentiated).

a Pills include daily combined and progestin-only oral contraceptives.

b Other modern methods include condoms, fertility-awareness-based methods (LAM, TwoDay Method, Standard Days Method) and emergency contraceptive pills.

**Table 3:** Predictive performance of full and simplified risk scores on contraceptive discontinuation at 24 weeks, by cohort

	Cohort		
Panel A. Full risk score	Derivation	Validation	Full
Proportion defined as “high risk”	41.8%	40.3%	41.4%
Sensitivity	70.9%	58.6%	67.9%
Specificity	78.6%	77.8%	78.3%
Positive predictive value	36.6%	31.4%	35.3%
Negative predictive value	93.9%	91.5%	93.3%

**Panel B. Simplified risk score**			
Proportion defined as “high risk”	63.4%	61.3%	62.9%
Sensitivity	80.6%	74.9%	79.2%
Specificity	57.1%	66.7%	60.9%
Positive predictive value	24.7%	28.1%	26.1%
Negative predictive value	94.4%	93.9%	94.4%

Notes: The full risk score includes the following variables: use of Cu-IUD, pills or other modern method (condoms or fertility-awareness-based methods) (vs. injectables or implants); switching methods or continuing a contraceptive method at clinic attendance (vs. newly initiating contraception); < 9 years of completed education; not having a child < 6 months of age; being unmarried or having a partner who is supportive of the participant's contraceptive use. The simplified risk includes only the following subset of variables: use of Cu-IUD, pills or other modern method (condoms or fertility-awareness-based methods) (vs. injectables or implants); switching methods or continuing a contraceptive method at clinic attendance (vs. newly initiating contraception); not having a child < 6 months of age and being unmarried. All estimates calculated for score cut point, defined as optimal based on maximizing Youden's *J* statistic. This corresponds to a score of > 6 for the full risk score and > 2 for the simplified risk score. All values calculated at 24 weeks.

A simplified risk score that excluded variables not routinely collected (spousal support for FP and educational attainment) had a median value of 3 points in the derivation cohort (IQR 2–3; range: 0–8) (Table 2). Performance of the simplified score was similar to the full score, with a 24-week AUC of 0.73 (95% CI 0.60–0.85) and 12-week AUC of 0.67 (95% CI 0.59–0.75) (Fig. 2A). The score demonstrated 80.6% sensitivity, 57.1% specificity and 24.7% PPV at the optimal cut point of 2, with 63.4% (*n* = 354) defined as high risk (Table 3). Discontinuation risk was significantly higher among women with scores of > 2 (vs. ≤ 2) (hazard ratio: 2.46, 95% CI 1.40–4.34) (Fig. 3A). Both risk scores demonstrated improved predictive ability over any single score component (Online Appendix).

### 3.2 Risk score validation

In the validation cohort, incidence of discontinuation was 35.9 per 100 woman-years (95% CI 24.0–53.5) (Fig. 1). The 24-week AUC was 0.68 (95% CI 0.47–0.90) for the full and 0.73 (95% CI 0.51–0.94) for the simplified risk score (Fig. 2B). The full risk score demonstrated 58.6% sensitivity and 77.8% specificity at the cut point defined in the derivation cohort, while the simplified score had 74.9% sensitivity and 66.7% specificity (Table 3). The risk scores demonstrated similar predictive ability in the full cohort (Table 3B, Online Appendix).

### 3.3 Sensitivity analyses and alternative methodological approaches

Overall, 60% (*n* = 45/75) of women reporting method discontinuation in the derivation cohort were missing reason for discontinuation (Online Appendix). To assess the possible impact of including discontinuation in our analyses due to desire for pregnancy, we conducted two sensitivity analyses: first, treating discontinuation for pregnancy desire as a competing risk; and second, additionally excluding women unsure of the preferred timing of next pregnancy. Both analyses yielded similar estimates as the primary model (Online Appendix). Alternative model selection using the LASSO-Cox approach resulted in a similar prediction model, selecting all variables from the full risk score and several additional variables (fertility intentions and perceived accuracy of information provided during FP visit), and predictive performance (Online Appendix). Our results were also robust to an alternative definition of discontinuation that required at least 4 weeks of nonuse and to a number of additional sensitivity analyses that assessed potential measurement error in self-reported method use (Online Appendix).

## 4 Discussion

All women seeking contraception would benefit from receiving accurate, culturally relevant and personalized counseling. However, a simple algorithm could be used to identify women seeking FP care who may benefit most from tailored counseling on strategies for method-related challenges to ensure that their contraceptive needs and preferences are met. We developed and assessed the validity of a pragmatic risk assessment tool in a cohort of Kenyan women seeking FP services to predict contraceptive discontinuation among women who do not wish to become pregnant. Using an IUD or short-term modern method, switching methods or continuing a specific method type (relative to newly initiating contraception), having < 9 years of completed education, not having a child < 6 months old, and being unmarried or having a spouse with supportive attitudes towards FP (versus a spouse who is unsupportive or whose attitudes are unknown) were selected as predictors in the full risk score. Both the full and simplified risk scores demonstrated moderate predictive ability to identify contraceptive discontinuation in the 24 weeks after receiving FP services but identified high proportions of women as high risk (> 40% and > 60% for the full and simplified score, respectively). While targeting “false positives” for additional counseling or support may not present additional risks to the patient, development of a score with improved positive predictive value is necessary to address pragmatic considerations regarding additional demands on provider time.

Among the variables selected for inclusion in our risk score, several are established risk factors for contraceptive discontinuation, including short-term method use, being unmarried and low educational attainment [7,11]. Relatively high discontinuation among IUD users was unexpected and may be explained in part by the small sample size. In a sensitivity analysis treating discontinuation for pregnancy desire as a competing risk, we observed a lower adjusted hazard of discontinuation among IUD users relative to implant and injectable users; this finding suggests that IUD users may be more likely to discontinue due to pregnancy desire. Further research in a larger sample that explores possible interactions between method type and future pregnancy intentions, planning and ambivalence is warranted. Surprisingly, we found that contraceptive discontinuation was higher among women who reported spousal support for FP. Perceived spousal attitudes may serve as a proxy measure for fertility intentions and attitudes towards a mistimed pregnancy [32,33]. In this study, we captured a single-item measure for pregnancy ambivalence which may have inadequately captured complex feelings towards pregnancy and parenthood [34]. Higher risk of discontinuation among method continuers, compared to switchers or initiators, may also be explained by unmeasured differences in the level and intensity of motivations to prevent pregnancy. Future studies may benefit from finer measurement of fertility intentions, pregnancy ambivalence and relationship dynamics [26].

Indicators of perceived quality of care and satisfaction were not selected for inclusion in the full risk score. Several recent studies have found that method information counseling reduced risk of discontinuation by 64%–80% [16,18]. However, these studies included women newly initiating or switching contraceptive methods; in contrast, 62% of our study participants were “continuers” at enrollment, and it is plausible that experienced contraceptive users may be less receptive to, or in need of, introductory information on topics such as alternative contraceptive method types and potential side effects.

A strength of this study is its prospective design and high-frequency data collection. The remote nature of data collection reduced participant burden, permitting weekly assessment of short-term and unintentional discontinuation events that may be missed in less frequent, retrospective contraceptive history-taking. Remote data collection may also reduce social desirability bias [35]. Women were recruited while seeking FP services in public facilities, and our estimates of discontinuation among women newly initiating a contraceptive method were similar to national data (25.8%) [36]. Facility-based recruitment also allowed for evaluation of indicators of perceived quality and satisfaction with FP services. Finally, we defined discontinuation based on 2 or more consecutive weeks of reported nonuse, rather than a single week, to reduce sensitivity to data entry errors.

Our study has several limitations. Measurement error may be higher in self-administered surveys given the absence of a trained enumerator to probe inconsistent responses, ensure comprehension and correct entry errors. While we did not validate self-reported contraceptive use using medical records or clinical assessment, future research would benefit from using supplementary data sources to validate self-reports. The SMS format encourages short survey instruments; we may therefore have failed to measure specific drivers of discontinuation including lack of sexual activity and experience of side effects at enrollment among continuers. Over half of all women who discontinued did not provide a reason, which limited our ability to differentiate method-related discontinuation from discontinuation for pregnancy desire. While censoring was not associated with method type, relatively high loss to follow-up may introduce selection bias. In addition, the number of IUD, pill and other modern method users was small, which limited our ability to explore predictors of discontinuation by method type and develop method-specific risk scores. The short duration of follow-up also makes our results generalizable only to early discontinuation events.

We developed and assessed validity of a pragmatic risk assessment tool to identify women at high risk of contraceptive discontinuation after utilization of FP services. The tool demonstrated moderate predictive ability but low positive predictive value. Future research is needed to develop provider-focused tools that can support women with contraceptive methods and care that are better aligned with their needs and preferences.

## Declaration of competing interests

The authors declare no competing interests.

## Funding

Research reported in this publication was supported by a Gates Grand Challenges Explorations grant (OPP1172004), the Eunice Kennedy Shriver National Institute of Child Health & Human Development of the National Institutes of Health (F31HD097841 [C.R.] and F32HD100202 [E.M.L.]) and the National Institute of Allergy and Infectious Diseases of the National Institutes of Health (K01AI11628) [A.L.D.]. The content is solely the responsibility of the authors and does not necessarily represent the official views of the National Institutes of Health.

## Appendix A Supplementary data

Supplementary material Image 1

## References

[1] Westoff C.F.D. (2006). New estimates of unmet need and the demand for family planning.

[2] Hancock N.L., Stuart G.S., Tang J.H., Chibwesha C.J., Stringer J.S.A., Chi B.H. (2016). Renewing focus on family planning service quality globally. Contracept. Reprod Med. 1. England.

[3] UNDP. Trends in contraceptive use worldwide. New York, New York: United Nations, Department of Economic and Social Affairs, Population Division; 2015. Contract no.: ST/ESA/SER.A/349.

[4] Gubrium A.C., Mann E.S., Borrero S., Dehlendorf C., Fields J., Geronimus A.T. (2016). Realizing reproductive health equity needs more than long-acting reversible contraception (LARC). Am J Public Health.

[5] Cleland J., Conde-Agudelo A., Peterson H., Ross J., Tsui A. (2012). Contraception and health. Lancet.

[6] Cleland J., Bernstein S., Ezeh A., Faundes A., Glasier A., Innis J. (2006). Family planning: the unfinished agenda. Lancet.

[7] Castle S, Askew I. Contraceptive discontinuation: reasons, challenges, and solution. Washington, D.C.: Population Council; 2015. Contract No.: 369641.

[8] Jain A., Obare F., RamaRao S., Askew I. (2013). Reducing unmet need by supporting women with met need. Int Perspect Sex Reprod Health.

[9] Ali M., Cleland J. (1995). Contraceptive discontinuation in six developing countries: a cause-specific analysis. Int Fam Plan Perspect.

[10] Raine T.R., Foster-Rosales A., Upadhyay U.D., Boyer C.B., Brown B.A., Sokoloff A. (2011). One-year contraceptive continuation and pregnancy in adolescent girls and women initiating hormonal contraceptives. Obstet Gynecol.

[11] Ali M., Cleland J., Shah I. (2012). Causes and consequences of contraceptive discontinuation: Evidence from 60 demographic and health surveys.

[12] Ali M., Cleland J. (1999). Determinants of contraceptive discontinuation in six developing countries. J Biosoc Sci.

[13] World Health Organization Department of Reproductive Health and Research (WHO/RHR), Johns Hopkins Bloomberg Center for Communication Programs (CCP) and the Knowledge for Health Project (KfHP). Family planning: a global handbook for providers (2018 update). Baltimore and Geneva: CCP and WHO; 2018.

[14] Dehlendorf C., Henderson J.T., Vittinghoff E., Grumbach K., Levy K., Schmittdiel J. (2016). Association of the quality of interpersonal care during family planning counseling with contraceptive use. Am J Obstet Gynecol.

[15] Jain A., Aruldas K., Mozumdar A., Tobey E., Acharya R. (2019). Validation of two quality of care measures: results from a longitudinal study of reversible contraceptive users in India. Stud Fam Plann.

[16] Chakraborty N.M., Chang K., Bellows B., Grepin K.A., Hameed W., Kalamar A. (2019). Association between the quality of contraceptive counseling and method continuation: findings from a prospective cohort study in social franchise clinics in Pakistan and Uganda. Glob Health Sci Pract.

[17] Mosepele M., Hemphill L.C., Palai T., Nkele I., Bennett K., Lockman S. (2017). Cardiovascular disease risk prediction by the American College of Cardiology (ACC)/American Heart Association (AHA) atherosclerotic cardiovascular disease (ASCVD) risk score among HIV-infected patients in sub-Saharan Africa. PLOS ONE..

[18] Jain A., Aruldas K., Tobey E., Mozumdar A., Acharya R. (2019). Adding a question about method switching to the method information index is a better predictor of contraceptive continuation. Global Health Sci Pract.

[19] Halpern V., Grimes D.A., Lopez L., Gallo M.F. (2006). Strategies to improve adherence and acceptability of hormonal methods for contraception. Cochrane Database Syst Rev.

[20] Castaño P.M., Bynum J.Y., Andrés R., Lara M., Westhoff C. (2012). Effect of daily text messages on oral contraceptive continuation: a randomized controlled trial. Obstet Gynecol..

[21] Canto De Cetina T.E., Canto P., Ordonez Luna M. (2001). Effect of counseling to improve compliance in Mexican women receiving depot-medroxyprogesterone acetate. Contraception.

[22] Pintye J., Drake A.L., Kinuthia J., Unger J.A., Matemo D., Heffron R.A. (2017). A risk assessment tool for identifying pregnant and postpartum women who may benefit from preexposure prophylaxis. Clin Infect Dis.

[23] Balkus J.E., Brown E., Palanee T., Nair G., Gafoor Z., Zhang J. (2016). An empiric HIV risk scoring tool to predict HIV-1 acquisition in African Women. J Acquir Immune Defic Syndr.

[24] Mateen F.J., Kanters S., Kalyesubula R., Mukasa B., Kawuma E., Kengne A.P. (2013). Hypertension prevalence and Framingham risk score stratification in a large HIV-positive cohort in Uganda. J Hypertens (Los Angel).

[25] Festin M.P., Kiarie J., Solo J., Spieler J., Malarcher S., Van Look P.F. (2016). Moving towards the goals of FP2020 — classifying contraceptives. Contraception.

[26] Tobey E., Jain A., Mozumdar A. (2020). The relationship between attitudes towards pregnancy and contraceptive continuation: results from a longitudinal study of married women in India. PLOS ONE.

[27] Zhang Z. (2016). Variable selection with stepwise and best subset approaches. Ann Transl Med.

[28] Blanche P., Dartigues J.-F., Jacqmin-Gadda H. (2013). Estimating and comparing time-dependent areas under receiver operating characteristic curves for censored event times with competing risks. Stat Med.

[29] Fine J.P., Gray R.J. (1999). A proportional hazards model for the subdistribution of a competing risk. J Am Stat Assoc.

[30] Azur M.J., Stuart E.A., Frangakis C., Leaf P.J. (2011). Multiple imputation by chained equations: what is it and how does it work?. Int J Methods Psychiatr Res.

[31] Sohn I., Kim J., Jung S.-H., Park C. (2009). Gradient lasso for Cox proportional hazards model. Bioinformatics.

[32] Higgins J.A., Hirsch J.S., Trussell J. (2008). Pleasure, prophylaxis and procreation: a qualitative analysis of intermittent contraceptive use and unintended pregnancy. Perspect Sex Reprod Health.

[33] Higgins J.A. (2017). Pregnancy ambivalence and long-acting reversible contraceptive (LARC) use among young adult women: a qualitative study. Perspect Sex Reprod Health.

[34] Gomez A.M., Arteaga S., Villasenor E., Arcara J., Freihart B. (2019). The misclassification of ambivalence in pregnancy intentions: a mixed-methods analysis. Perspect Sex Reprod Health.

[35] Barden-O’Fallon J., Speizer I. (2011). What differentiates method stoppers from switchers? Contraceptive discontinuation and switching among Honduran women. Int Perspect Sex Reprod Health.

[36] Kenya National Bureau of S, Ministry of HK, National ACCK, Kenya Medical Research I, National Council for Population and DK. Kenya demographic and health survey 2014. Rockville, MD, USA; 2015.

