# Supplementary material for: A risk scoring tool for predicting Kenyan women at high risk of contraceptive discontinuation
Source: Contracept X. 2020 Oct 29;2:100045. doi: 10.1016/j.conx.2020.100045 (PMC7683324; doi:10.1016/j.conx.2020.100045)

**A Risk Scoring Tool for Predicting Women at High Risk of Contraceptive Discontinuation:**

**Supplementary Materials**

1. **Method ascertainment over follow up**

Contraceptive use was ascertained weekly via self-reported response to the question, “Are you currently using any method of family planning?” For affirmative responses, respondents were asked to provide the current method type. To assess use of fertility-based methods, women were given the option to select use of a “natural method,” terminology which was developed with the Kenya-based study team. Fertility awareness-based (FAB) method users were then asked to specify use of “counting days” or “breastfeeding method (before return of periods after childbirth)”. Because study eligibility required use of modern FAB methods, women reporting “natural method” use over follow-up were assumed to be using modern contraception in our primary analyses. To ascertain use of emergency contraceptive pills (ECP), women reported that they were currently using contraceptive pills and subsequently specified the type as “e-pill.” Women using coitus-dependent methods were not asked about contraceptive method use relative to sexual activity.

We assessed practice of the lactational amenorrhea method (LAM) through the following questions: "Which method are you currently using? Natural method. Which natural method are you currently using? Breastfeeding method (before return of periods after childbirth)." To be consistent with our treatment of use of other methods, which was only based on reported use rather than on specifics of adherence or method eligibility, we did not restrict reported LAM use only to those who meet the eligibility criteria.

1. **Method-specific considerations for defining discontinuation**

***Depot medroxyprogesterone (DMPA)***

While participants in our study did not specify type of injectable used, DMPA is the primary and typically only injectable method available in Kenya’s public sector. DMPA has an established duration of efficacy of 13-17 weeks. In our primary analyses, we allow earlier instances of discontinuation among injectable users based on their self-reported use of no method. While this introduces probable measurement error among DMPA users in the discontinuation outcome, we selected this as our primary approach because no similar restrictions (such as a 17 week window of “immunity” from discontinuation) could be applied to users of other method types. We did not correct DMPA discontinuations based on scientific knowledge in the primary analysis, based on our expectation that this would introduce differential measurement error by method type (as similar corrections could not be made for other method users who likely also incorrectly reported non-use in some cases).

We assessed the robustness of our findings in an sensitivity analysis that prohibited DMPA discontinuation within 17 weeks of the most recent reported injection (Tables S7 and S8). Restricting discontinuation to at least 17 weeks after the most recent injection reduces the estimated incidence rate of discontinuation among baseline injectable users considerably, from 35.0 discontinuations per 100 woman-years (95% CI 25.6, 48.0) in the primary analysis to 11.7 per 100 woman-years (95% CI 6.9, 19.8). However, we found that the prediction model selected using data in which we restrict early DMPA discontinuation is very similar to that selected by our primary approach. Comparing the full risk score in Table 2 to Table S8, we find that all predictors from our primary model are selected using the alternative DMPA discontinuation definition and that estimated coefficients are these predictors are qualitatively and quantitatively similar across specifications. These findings indicate that, while early discontinuations may artificially inflate estimated incidence of discontinuation among injectable users, predictors of discontinuation are relatively stable.

***Coitus-dependent methods (ECP, condom, and FAB)***

Due to the required brevity of the weekly surveys, we did not capture information on sexual activity over follow-up. By asking about women’s “current method of family planning” each week, our intention was to capture use of coitus-dependent methods even in the absence of sexual activity. However, it is possible that coitus-dependent method users may be incorrectly categorized as discontinuers due to lack of sexual activity in the prior week.

We assess robustness of the risk score to this potential measurement error in several sensitivity analyses that exclude coitus-dependent and FAB methods (Table S9 and S10). In sub-groups excluding ECP and FAB method users (Table S9) and additionally excluding condom users (Table S10), we find that predictor models based on the primary stepwise AIC selection method are very similar to those developed in the full cohort (presented in Table 2). Predictors selected in both subgroups include all those selected in the primary model, with similar estimated coefficients.

***Short-term gaps in method use***

We deliberately selected a short (2-week) interval of method non-use to define our primary definition of contraceptive discontinuation. This decision was made in order to examine short-term or even unintentional lapses in method use that may nevertheless be meaningful discontinuation events in terms of increased risk of unintended pregnancy. However, we acknowledge that pregnancy risk during short-term gaps in method use differs by method type. We therefore conducted a sensitivity analysis in which we calculated incidence rates by FP method and user type at enrollment (Table S11) and rederived the risk score (Table S12) using an alternate definition of discontinuation based on a 4- rather than 2-week gap in modern method use. We find that, while the alternate definition results in lower rates of discontinuation, the risk score development remains relatively robust to definition changes: IUD and short-term modern method use, <9 years completed education, not having a child aged <6 months, and either being unmarried or having a husband who is supportive of FP are selected as predictive factors in the risk score model with associations of similar magnitude as the primary model. One additional metric of patient satisfaction (being less than “very satisfied” with the FP services received) is also identified as a predictor of discontinuation.

1. **Treatment of missing data**

For observations missing data on method use or current method type over follow-up, we used a hybrid of last observation carried forward (LOCF) and next observation carried backward (NOCB) to singly impute method type (including use of no method) to the midpoint of the missing interval. Participants were censored at their latest observed follow-up time, with no attempt made to impute monotonic right censoring after the final complete follow-up observation. We made no attempt to impute missing baseline covariates.

***Descriptive summary of missing data over follow-up, by FP user and method type***

Among participants with complete baseline covariates of interest (n=835), we observe 744/835 (89%) had some follow-up and were included in the full analytic cohort. We compared differences in mean inclusion in the analysis by FP user type and method type used at study enrollment using Wald tests of means. We find no evidence of exclusion by method type. However, method switchers were slightly less likely to have any completed follow-up compared to initiators (90% vs. 84%, p=0.04).

The average completed follow-up in the full cohort included in the analysis (N=744) was 20.5 weeks. We observe no differences in righthand censoring by method or FP user type among those included in the full analytic cohort. Overall, 3,194/15,266 (21%) of weekly observations in the full cohort were imputed to account for interval censoring. Compared to injectable users, we observe no differences in the proportion of each participant's follow-up observations that were imputed by method category with the exception of Cu-IUD users, who had fewer censored observations (17% vs. 25%, p=0.003). Compared to initiators, method continuers had a higher proportion of imputed observations (24% vs. 19%, p=0.004), but no difference in interval censoring was found between initiators and switchers.

1. **Time-dependent receiver operating curves (ROC) and area under the curve (AUC) analysis**

We calculated time-dependent ROC-AUC, sensitivity, specificity, and positive and negative predictive values using the nonparametric inverse probability of censoring weighting (IPCW) estimator proposed by Blanche et al. and operationalized using the *timeROC* package for R, version 0.4 (<https://cran.r-project.org/web/packages/timeROC/timeROC.pdf>). ROC-AUC analyses were specified at 84 days (12 weeks) and 168 days (24 weeks). The Kaplan-Meier estimator was used to calculate censoring weights. Sensitivity and specificity were at the optimal cut-point for the full and simplified risk scores at time equal to 168 days.

1. **Group LASSO-Cox model specification**

***Cox proportional hazards model***

We used the Efron approach to handle ties in all Cox Proportional Hazards models.

***Group LASSO-cox model***

The group Cox-LASSO model with the smallest mean cross-validation error was selected using 100 repeated 10-fold cross-validations. Coefficients for variables selected using the group Cox-LASSO approach were estimated using a standard Cox model.

1. **Cross-validation of ROC-AUC performance metrics**

Cross-validation of the 24-week AUC and Brier scores were estimated using the “resample” function of the *MachineShop* package for R, version 2.1.0 (<https://cran.r-project.org/web/packages/MachineShop/MachineShop.pdf>). Performance metrics were calculated specifying a Cox model with the dependent variable equal to the continuous risk score (full, simplified and Cox LASSO), using the Efron method for ties.

1. **Consideration of FP users who did not receive FP services on the date of study enrollment**

Participants were recruited from FP and MCH clinics within public health facilities and were eligible if they were currently using a modern contraceptive method. Eligibility was not contingent on receipt of FP services on the date of enrollment. As a result, 12% (89/744) of the analytic cohort reported most recently receiving FP services more than 1 day +/- the recorded enrollment date. As a result, “continuers” may comprise two distinct profiles of contraceptive users: 1) women seeking FP services on the date of enrollment in order to obtain method resupply, for a method-related check-up, or to obtain counseling or care related to contraceptive use; and 2) women seeking other MCH services who also happened to be using a modern contraceptive method. We conducted a sensitivity analysis to assess robustness of our findings to exclusion of women who reported that their most recent FP visit was not within 1 day of study enrollment (Table S13). Rederiving the prediction model in this restricted sample (N=449) resulted in similar predictor selection: IUD and short-term modern method use, <9 years completed education, not having a child aged <6 months, and either being unmarried or having a husband who is supportive of FP are selected as predictive factors in the risk score model with associations of similar magnitude as the primary model. Two additional predictors were also selected: a travel time of less than 30 minutes to the health facility and receiving more elements of method information counseling were both included as predictors based on the minimum AIC criterion, although neither of these predictors was associated with risk of discontinuation at the p=0.05 level. These findings indicate that our model is robust to inclusion of modern method users who were recruited for study participant while seeking health services other than FP care.

**Tables**

**Table S1. Characteristics of the included and excluded sample**

|  | | | |  |
| --- | --- | --- | --- | --- |
|  | **Included in Analysis (n=744)** | **Excluded from Analysis* (n=468)** | |  |
|  | n (%) | n | n (%) | **p** |
| **Sociodemographic characteristics** |  |  |  |  |
| Age (years) [median (IQR)] | 26 (23, 31) | 363 | 37 (23, 31) | 0.54 |
| Completed education <9 years | 376 (51) | 385 | 236 (61) | 0.001 |
| *Relationship status* |  |  |  |  |
| Not married (legal or presumed) | 139 (19) | 363 | 67 (18) | 0.89 |
| Partner supportive of FP | 561 (75) |  | 277 (76) |  |
| Partner not supportive of FP or unsure of partner support | 44 (6) |  | 19 (5) |  |
| **Reproductive characteristics** |  |  |  |  |
| Number of living children [median (IQR)] | 2 (1, 3) | 317 | 2 (1, 3) | 0.29 |
| Does not have a child aged <6 months | 524 (70) | 317 | 238 (75) | 0.12 |
| *Fertility intentions* |  |  |  |  |
| Unsure intention to have children or unsure of preferred timing | 147 (20) | 322 | 77 (24) | 0.001 |
| Desires no future children | 159 (21) |  | 93 (29) |  |
| Desires next pregnancy in 1-2 years | 67 (9) |  | 33 (10) |  |
| Desires next pregnancy in >2 years | 371 (50) |  | 119 (37) |  |
| *Pregnancy in the short-term future would be a:* |  |  |  |  |
| Not sure | 99 (13) | 322 | 47 (15) | 0.54 |
| Big problem | 413 (56) |  | 163 (51) |  |
| Small problem | 63 (8) |  | 30 (9) |  |
| No problem | 169 (23) |  | 82 (25) |  |
| **Characteristics of FP services received** |  |  |  |  |
| Contraceptive method type |  |  |  |  |
| Injectables | 310 (42) | 308 | 108 (35) | <0.001 |
| Implant | 320 (43) |  | 122 (40) |  |
| Intrauterine device (IUD) | 46 (6) |  | 21 (7) |  |
| Pills^a^ | 36 (5) |  | 23 (7) |  |
| Other modern^b^ | 32 (4) |  | 34 (11) |  |
| FP user type |  |  |  |  |
| Initiating contraception | 194 (26) | 304 | 88 (29) | 0.24 |
| Switching from one method type to another | 91 (12) |  | 27 (9) |  |
| Continuing method used in past month | 459 (62) |  | 189 (62) |  |
| History of contraceptive side effects |  |  |  |  |
| No | 384 (52) | 282 | 132 (47) | 0.06 |
| Yes | 349 (47) |  | 140 (50) |  |
| Unsure | 11 (1) |  | 10 (4) |  |
| Traveled less than 30 minutes to reach health facility | 469 (63) | 286 | 183 (64) | 0.78 |
| **Quality of care and satisfaction** |  |  |  |  |
| "Very satisfied" with services received | 353 (47) | 284 | 122 (43) | 0.20 |
| Felt that her privacy was not protected during the visit | 47 (6) | 283 | 14 (5) | 0.41 |
| Felt provider gave accurate information | 679 (91) | 278 | 247 (89) | 0.24 |
| Felt provider's treatment was "very respectful" | 653 (88) | 280 | 233 (83) | 0.06 |
| Number of items in Method Information Index received [median (IQR)]^c^ | 3 (1, 3) | 261 | 3 (1, 3) | 0.70 |
| Feelings about using FP: |  |  |  |  |
| No fears or concerns | 48 (65) | 320 | 181 (57) | 0.007 |
| Reported having fears or concerns | 239 (32) |  | 117 (37) |  |
| Unsure of having fears or concerns | 25 (3) |  | 2 (7) |  |
| Notes: p-values generated using Chi-squared tests for categorical variables and t-tests of means assuming unequal variance for continuous variables. | | | | |
| ^a^ Pills include daily combined and progestin-only oral contraceptives  ^b^ Other modern methods include condoms, fertility-awareness based methods (LAM, Two Days Method, Standard Days Method), and emergency contraceptive pills  ^c^ The Method Information Index is calculated based on 3 questions: "during your visit: 1) were you informed about other methods?, 2) were you informed about side effects or problems with the method?, 3) were you told what to do if you had side effects of problems with the method?". Responses were summed to provide a count of the number of counseling items received, from 0 (received none of these counseling items) to 3 (received all items). * Women were excluded due to missing values in any baseline characteristics considered as risk factors or due to completely missing follow-up data. Reported characteristics are based on complete observations within each variable, with no imputation of missing values. Sample sizes for each variable are provided to show level of missingness within individual characteristics in the excluded sample. | | | | |

**Table S2. AUC Estimates for Individual Risk Score Components**

|  | **AUC (95% CI)** | |
| --- | --- | --- |
|  | ***Derivation Cohort*** | ***Validation Cohort*** |
| ***Contraceptive method type*** |  |  |
| IUD | 0.59 (0.48, 0.70) | 0.52 (0.40, 0.65) |
| Pills^a^ |  |  |
| Other modern^b^ |  |  |
| **FP user type** |  |  |
| Continuing method used in past month | 0.50 (0.40, 0.60) | 0.51 (0.35, 0.67) |
| Switching from one method type to another |  |  |
| <9 years completed education | 0.62 (0.48, 0.76) | 0.45 (0.26, 0.64) |
| Does not have child aged <6 months | 0.61 (0.48, 0.75) | 0.79 (0.63, 0.96) |
| ***Relationship status*** |  |  |
| Partner supportive of FP | 0.49 (0.48, 0.51) | 0.50 (0.50, 0.50) |
| Not married (legal or presumed) |  |  |
| ^a^ Pills include daily combined and progestin-only oral contraceptives  ^b^ Other modern methods include condoms, fertility-awareness based methods (LAM, Two Days Method, Standard Days Method), and emergency contraceptive pills  Notes: AUC-ROC at time equal to 24 weeks are presented for univariate Cox PH models. For categorical variables, levels within the categorical variable were modeled jointly. | | |

**Table S3. Sensitivity, specificity, and Youden’s J statistic at selected cut point values**

|  | **Sensitivity** | **Specificity** | **Youden's J statistic** |
| --- | --- | --- | --- |
| Full risk score |  |  |  |
| 5 | 0.857 | 0.429 | 0.286 |
| 6 | 0.709 | 0.786 | 0.495 |
| 7 | 0.332 | 0.929 | 0.261 |
| Simplified risk score |  |  |  |
| 1 | 0.934 | 0.143 | 0.077 |
| 2 | 0.806 | 0.571 | 0.378 |
| 3 | 0.425 | 0.857 | 0.282 |
| Group-LASSO risk score | |  |  |
| 14 | 0.828 | 0.429 | 0.256 |
| 15 | 0.761 | 0.786 | 0.546 |
| 16 | 0.658 | 0.857 | 0.515 |
| Notes: Time-dependent sensitivity and specificity estimates were calculated at 24 weeks. Youden's J statistic is defined as sensitivity + specificity - 1. The optimal cut point is selected as the value with the highest J statistic value. | | | |

**Table S4. Cross-validated ROC-AUC and Brier scores at 24 weeks, by risk score**

|  | **Full Cohort** | |
| --- | --- | --- |
| ***Risk Score*** | ***cvAUC*** | ***Brier Score*** |
| Full | 0.70 (0.58, 0.81) | 0.14 (0.09, 0.19) |
| Simplified | 0.67 (0.54, 0.78) | 0.14 (0.09, 0.19) |
| Group-LASSO | 0.71 (0.60, 0.83) | 0.14 (0.09, 0.19) |
| Notes: 95% CI in parentheses. Cross-validated AUC and Brier Scores estimated using repeated 5-fold cross-validation with 100 repeats in the full cohort. | | |

**Table S5. Sensitivity analyses: Estimated coefficients and risk score calculations for restricted and competing-risk models**

|  | **Analysis 1*** | **Analysis 2†** |
| --- | --- | --- |
|  | aSHR | aHR |
| ***Contraceptive method type*** |  |  |
| IUD | 0.77 | 1.43 |
| Pills^a^ | 2.48 | 3.31 |
| Other modern^b^ | 6.75 | 5.35 |
| *Reference: Injectables, implants, or* Cu-IUD/IUS | *Ref.* | *Ref.* |
| ***FP user type*** |  |  |
| Continuing method used in past month | 2.27 | 2.29 |
| Switching from one method type to another | 2.34 | 1.72 |
| *Ref: Initiating contraception* | *Ref.* | *Ref.* |
| <9 years completed education | 2.01 | 1.68 |
| Does not have child aged <6 months | 1.64 | 1.37 |
| ***Relationship status*** |  |  |
| Partner supportive of FP | 3.59 | 3.96 |
| Not married (legal or presumed) | 6.30 | 8.42 |
| *Reference: partner unsupportive/unsure of partner support* | *Ref.* | *Ref.* |
| * In sensitivity analyses 1, we estimate adjusted subhazards ratios for the components of the full risk score selected in the primary analysis but using a competing-risks survival model, with discontinuation due to desire to become pregnant considered a competing risk. We used multiple imputation with chained equations and 10 imputed datasets to model missing reasons for discontinuation. | | |
| † In sensitivity analysis 2, we exclude 68 women in the derivation cohort who reported a desire for a pregnancy in the future but who were unsure of their preferred timing (total sample in restricted derivation cohort of N=490). We report adjusted hazard ratios.  ^a^ Pills include daily combined and progestin-only oral contraceptives  ^b^ Other modern methods include condoms, fertility-awareness based methods (LAM, Two Days Method, Standard Days Method), and emergency contraceptive pills | | |

**Table S6. Sensitivity analyses: estimated Cox model coefficients and risk score calculations using alternate methodological approach**

|  | **Group LASSO Risk Score (n=558)** | |
| --- | --- | --- |
|  | **β** | **Points** |
| ***Contraceptive method type*** |  |  |
| Cu-IUD/IUS | 0.40 | 2 |
| Pills^a^ | 1.09 | 4 |
| Other modern^b^ | 1.65 | 7 |
| *Reference: implant, injectables* | *Ref.* | 0 |
| ***Reason for FP visit:*** |  |  |
| Continuing method used in past month | 0.75 | 3 |
| Switching from one method type to another | 0.79 | 3 |
| *Ref: initiating contraception* | *Ref.* | 0 |
| ***Fertility intentions*** |  |  |
| Does not desire future pregnancy | 0.61 | 3 |
| Desires next pregnancy in 1-2 years | 0.44 | 2 |
| Desires next pregnancy in >2 years | 0.24 | 1 |
| *Ref: Not sure if desires future pregnancy or unsure of preferred timing* | *Ref.* | 0 |
| Reported information provided was accurate | 0.73 | 3 |
| <9 years of completed education | 0.56 | 2 |
| Does not have child aged <6 months | 0.44 | 2 |
| ***Relationship status*** |  |  |
| Partner supportive of FP | 1.38 | 6 |
| Not married (legal or presumed) | 1.93 | 8 |
| *Reference: partner unsupportive/unsure of partner support* | *Ref.* | 0 |
| **Maximum score** |  | **28** |
| Notes: In the group LASSO model, variable selection was conducted using the group LASSO model to identify variables with non-zero coefficients; for the risk score point calculation, coefficients were estimated using a standard Cox PH model.  ^a^ Pills include daily combined and progestin-only oral contraceptives  ^b^ Other modern methods include condoms, fertility-awareness based methods (LAM, Two Days Method, Standard Days Method), and emergency contraceptive pills | | |

**Table S7. Sensitivity analysis: Incidence rate of discontinuation in the full cohort, comparing primary analysis to one that restricts DMPA discontinuation based on established duration of efficacy**

|  | **Primary analysis** | **DPMA sensitivity analysis^a^** |
| --- | --- | --- |
|  | IR (95% CI)  per 100 woman-years | IR (95% CI)  per 100 woman-years |
| ***Contraceptive method type at enrollment*** | |  |
| Injectables | 35.0 (25.6, 48.0) | 11.7 (6.9, 19.8) |
| ^a^ In this sensitivity analysis, injectable users at enrollment could not experience a discontinuation event until at least 17 weeks since the last injection date reported. We assume that all injectable users were using DMPA, although injectable type was not captured in the surveys and it may be possible that other injectable contraceptives with shorter windows of efficacy were used. Most recent injection date was updated over time for individuals who continued injectable use or switched to injections over follow-up. If the most recent injection date was missing at baseline, we treated the most recent injection date as equal to the enrollment date. Notes: IR indicates the incidence rate of method discontinuation in the full cohort (n=774). 95% confidence intervals were estimated using Stata's native "stptime" command. | | |

**Table S8. Sensitivity analysis: predictors of discontinuation in derivation cohort, restricting discontinuation among injectable users to ≥17 weeks since most recent injection**

|  | **Full List of Predictors** | **Simplified Predictors** |
| --- | --- | --- |
|  | **HR (95% CI)** | **HR (95% CI)** |
| ***Contraceptive method type*** |  |  |
| Implant | 1.65 (0.81, 3.34) | 1.62 (0.81, 3.25) |
| Cu-IUD | 2.99 (1.10, 8.11) | 3.14 (1.18, 8.39) |
| Pills^a^ | 6.39 (2.74, 14.91) | 7.11 (3.10, 16.36) |
| Other modern^b^ | 12.11 (4.49, 32.68) | 9.39 (3.69, 23.92) |
| *Reference: Injectables* | *Ref.* | *Ref.* |
| ***FP user type*** |  |  |
| Continuing method used in past month | 3.70 (1.49, 9.22) | 3.75 (1.55, 9.10) |
| Switching from one method type to another | 3.14 (1.08, 9.13) | 3.29 (1.15, 9.47) |
| *Reference: Initiating contraception* | *Ref.* | *Ref.* |
| ***Fertility intentions*** |  |  |
| Desires no future children | 3.54 (1.31, 9.59) | -- |
| Desires next pregnancy in 1-2 years | 1.92 (0.63, 5.87) | -- |
| Desires next pregnancy in >2 years | 1.72 (0.72, 4.13) | -- |
| *Ref. Unsure intention to have children or unsure of preferred timing* | *Ref.* | *--* |
| **<9 years completed education** | 1.73 (0.99, 3.03) | -- |
| **Does not have child aged <6 months** | 2.08 (0.98, 4.42) | 1.94 (0.93, 4.02) |
| **Number of living children** | 0.81 (0.63, 1.04) | -- |
| ***Relationship status*** |  |  |
| Partner supportive of FP | 2.78 (0.38, 20.52) | -- |
| Not married (legal or presumed) | 5.51 (0.71, 42.77) | 1.88 (1.00, 3.51) |
| *Reference: partner unsupportive/unsure of partner support* | *Ref.* | -- |
| **Traveled less than 30 minutes to reach health facility** | 2.48 (1.33, 4.64) | -- |
| ^a^ Pills include daily combined and progestin-only oral contraceptives  ^b^ Other modern methods include condoms, fertility-awareness based methods (LAM, Two Days Method, Standard Days Method), and emergency contraceptive pills Notes: In this sensitivity analysis, injectable at enrollment could not experience a discontinuation event until at least 13 weeks since the last injection (assumed to be DMPA) reported at baseline. If the most recent injection date was missing, we treated the most recent injection date as equal to the enrollment date. Predictors selected using stepwise forwards and backwards to identify the Cox model with the minimum AIC value. The Efron approach was used to handle ties. The simplified model was developed by removing predictors identified in the full model that are not routinely collected (either verbally or in written documentation) in Kenyan public health facilities. | | |

**Table S9.** **Sensitivity analysis: predictors of discontinuation in derivation cohort, excluding women using emergency contraceptive pills or fertility-based methods at enrollment (N=546)**

|  | **Full List of Predictors** | **Simplified Predictors** |
| --- | --- | --- |
|  | **HR (95% CI)** | **HR (95% CI)** |
| ***Contraceptive method type*** |  |  |
| Cu-IUD/IUS | 1.52 (0.65, 3.56) | 1.55 (0.66, 3.65) |
| Pills^a^ | 3.09 (1.56, 6.12) | 3.33 (1.68, 6.60) |
| Condoms^b^ | 5.03 (1.78, 14.26) | 5.65 (1.98, 16.06) |
| *Reference: Injectables, implants* | *Ref.* | *Ref.* |
| ***FP user type*** |  |  |
| Continuing method used in past month | 2.11 (1.07, 4.18) | 2.23 (1.13, 4.40) |
| Switching from one method type to another | 2.01 (0.81, 4.96) | 1.92 (0.78, 4.73) |
| *Reference: Initiating contraception* | *Ref.* | *Ref.* |
| **<9 years completed education** | 1.63 (1.00, 2.66) | -- |
| **Does not have child aged <6 months** | 1.63 (0.89, 2.98) | 1.59 (0.87, 2.92) |
| ***Relationship status*** |  |  |
| Partner supportive of FP | 3.91 (0.54, 28.47) | -- |
| Not married (legal or presumed) | 6.40 (0.85, 48.25) | 1.70 (0.98, 3.00) |
| *Reference: partner unsupportive/unsure of partner support* | *Ref.* | -- |
| Felt provider gave accurate information | 2.34 (0.72, 7.54) | -- |
| ^a^ Pills include daily combined and progestin-only oral contraceptives  ^b^ In this analysis, all users of fertility-based or emergency contraception which are included in the "other modern methods" category in the primary analyses are excluded for total N=546 in the derivation cohort. Notes: Predictors selected using stepwise forwards and backwards to identify the Cox model with the minimum AIC value. The Efron approach was used to handle ties. The simplified model was developed by removing predictors that are not routinely collected (either verbally or in written documentation) in Kenyan public health facilities. | | |

**Table S10. Sensitivity analysis: predictors of discontinuation in derivation cohort, excluding women using condoms, emergency contraceptive pills, or fertility-based methods (N=534)**

|  | **Full List of Predictors** | **Simplified Predictors** |
| --- | --- | --- |
|  | **HR (95% CI)** | **HR (95% CI)** |
| ***Contraceptive method type*** |  |  |
| Cu-IUD/IUS | 1.55 (0.66, 3.63) | 1.56 (0.66, 3.67) |
| Pills^a^ | 3.10 (1.56, 6.14) | 3.34 (1.69, 6.62) |
| *Reference: Injectables, implants* | *Ref.* | *Ref.* |
| ***FP user type*** |  |  |
| Continuing method used in past month | 1.94 (0.96, 3.91) | 2.10 (1.04, 4.22) |
| Switching from one method type to another | 1.93 (0.78, 4.80) | 1.81 (0.73, 4.49) |
| *Reference: Initiating contraception* | *Ref.* | *Ref.* |
| **<9 years completed education** | 1.95 (1.17, 3.26) | -- |
| **Does not have child aged <6 months** | 1.54 (0.83, 2.86) | 1.48 (0.79, 2.75) |
| ***Relationship status*** |  |  |
| Partner supportive of FP | 3.94 (0.54, 28.76) | -- |
| Not married (legal or presumed) | 6.52 (0.86, 49.25) | 1.70 (0.97, 3.00) |
| *Reference: partner unsupportive/unsure of partner support* | *Ref.* | -- |
| Felt provider gave accurate information | 2.44 (0.76, 7.87) | -- |
| ^a^ Pills include daily combined and progestin-only oral contraceptives  Notes: Restricted sample of derivation cohort excluded all users of condoms, emergency contraceptive pills, and fertility-based methods, for a total sample size of N=534. Predictors selected using stepwise forwards and backwards to identify the Cox model with the minimum AIC value. The Efron approach was used to handle ties. The simplified model was developed by removing predictors that are not routinely collected (either verbally or in written documentation) in Kenyan public health facilities. | | |

**Table S11. Sensitivity analysis: Incidence rate of discontinuation in the full cohort (n=744), comparing 2 and 4 week intervals of method non-use to define discontinuation**

| **Discontinuation defined as:** | | |
| --- | --- | --- |
|  | **≥2 weeks of no modern method use** | **≥4 weeks of no modern method use** |
|  | IR (95% CI)  per 100 woman-years | IR (95% CI)  per 100 woman-years |
| **Contraceptive method type** |  |  |
| Injectables | 35.0 (25.6, 48.0) | 19.2 (12.6, 29.2) |
| Implants | 26.8 (19.0, 37.9) | 11.4 (6.7, 19.2) |
| Pills^a^ | 104.2 (59.2, 183.5) | 56.5 (26.9, 118.4) |
| Cu-IUD | 41.0 (19.6, 86.1) | 22.4 (8.4, 59.6) |
| Other modern^b^ | 100.2 (52.1, 192.6) | 60.5 (27.2, 134.8) |
| **FP user and contraceptive method type** |  |  |
| ***Initiating modern contraception*** | 22.0 (13.5, 35.9) | 14.8 (8.2, 26.7) |
| Injectables | 32.9 (16.4, 65.7) | 28.3 (13.5, 59.3) |
| Implants | 16.5 (7.4, 36.7) | 5.4 (1.3, 21.4) |
| Pills^a^ | 0.0 (--, --)± | 0.0 (--, --)± |
| Cu-IUD | 0.0 (--, --)± | 0.0 (--, --)± |
| Other modern^b^ | 60.2 (15.1, 240.6) | 56.6 (14.2, 226.3) |
| ***Switching from one method type to another*** | 34.1 (18.9, 61.5) | 14.9 (6.2, 35.8) |
| Injectables | 35.1 (8.8, 140.3) | 17.4 (2.4, 123.3) |
| Implants | 30.4 (13.6, 67.6) | 9.7 (2.4, 38.7) |
| Pills^a^ | 105.3 (26.3, 421.2) | 105.3 (26.3, 421.2) |
| Cu-IUD | 0 (--, --)± | 0 (--, --)± |
| Other modern^b^ | 186.2 (26.2, 1322.0) | 0 (--, --)± |
| ***Continuing method used in past month*** | 44.1 (35.0, 55.6) | 21.8 (15.8, 30.1) |
| Injectables | 35.7 (24.8, 51.4) | 16.7 (9.9, 28.1) |
| Implants | 63.2 (20.4, 49.0) | 15.4 (8.3, 28.6) |
| Pills^a^ | 145.0 (78.0, 269.5) | 64.3 (26.8, 154.4) |
| Cu-IUD | 104.2 (49.7, 218.6) | 53.0 (19.9 141.3) |
| Other modern^b^ | 117.2 (52.6, 260.8) | 73.2 (27.5, 195.0) |
| ^a^ Pills include daily combined and progestin-only oral contraceptives  ^b^ Other modern methods include condoms, fertility-awareness based methods (LAM, Two Days Method, Standard Days Method), and emergency contraceptive pills ± Confidence intervals not calculated, due to 0 observed failures in this group. Notes: IR indicates the incidence rate of method discontinuation. 95% confidence intervals were estimated using Stata's native "stptime" command. | | |

**Table S12. Sensitivity analysis: predictors of discontinuation in derivation cohort, with discontinuation defined as at least 4 weeks of no method use**

|  | **Full List of Predictors** | **Simplified Predictors** |
| --- | --- | --- |
|  | **HR (95% CI)** | **HR (95% CI)** |
| ***Contraceptive method type*** |  |  |
| Cu-IUD/IUS | 1.87 (0.65, 5.38) | 1.82 (0.64, 4.22) |
| Pills^a^ | 4.03 (1.74, 9.35) | 4.25 (1.84, 9.81) |
| Other modern^b^ | 5.95 (2.22, 15.91) | 4.86 (1.86, 12.70) |
| *Reference: Injectables, implants* | *Ref.* | *Ref.* |
| **<9 years completed education** | 1.55 (0.82, 2.93) | -- |
| **Does not have child aged <6 months** | 2.19 (0.96, 4.99) | 2.17 (0.96, 4.95) |
| ***Relationship status*** |  |  |
| Partner supportive of FP | 2.16 (0.29, 15.95) | -- |
| Not married (legal or presumed) | 4.80 (0.62, 36.91) | 2.00 (1.04, 3.86) |
| *Reference: partner unsupportive/unsure of partner support* | *Ref.* | -- |
| **Less than "very satisfied" with FP services received** | 1.80 (0.92, 3.51) | -- |
| ^a^ Pills include daily combined and progestin-only oral contraceptives  ^b^ Other modern methods include condoms, fertility-awareness based methods (LAM, Two Days Method, Standard Days Method), and emergency contraceptive pills Notes: In this analysis, we use an alternative definition of the discontinuation outcome that requires 4 weeks of non-use of any modern method (rather than the 2 week period required in the primary definition). Predictors selected using stepwise forwards and backwards to identify the Cox model with the minimum AIC value. The Efron approach was used to handle ties. The simplified model was developed by removing predictors identified in the full model that are not routinely collected (either verbally or in written documentation) in Kenyan public health facilities. | | |

**Table S13. Sensitivity analysis: predictors of discontinuation in derivation cohort, excluding women who did not receive FP services at enrollment (N=449)**

|  | **Full List of Predictors** | **Simplified Predictors** |
| --- | --- | --- |
|  | **HR (95% CI)** | **HR (95% CI)** |
| ***Contraceptive method type*** |  |  |
| Cu-IUD/IUS | 1.40 (0.50, 3.92) | 1.39 (0.50, 3.92) |
| Pills^a^ | 3.08 (1.50, 6.34) | 3.17 (1.54, 6.52) |
| Other modern^b^ | 4.89 (2.13, 11.21) | 4.38 (1.95, 9.84) |
| *Reference: Injectables, implants* | *Ref.* | *Ref.* |
| ***FP user type*** |  |  |
| Continuing method used in past month | 2.36 (1.15, 4.87) | 2.31 (1.13, 4.71) |
| Switching from one method type to another | 1.78 (0.71, 4.48) | 1.85 (0.74, 4.66) |
| *Reference: Initiating contraception* | *Ref.* | *Ref.* |
| **<9 years completed education** | 1.74 (1.02, 2.99) | -- |
| **Does not have child aged <6 months** | 1.72 (0.91, 3.26) | 1.67 (0.89, 3.13) |
| ***Relationship status*** |  |  |
| Partner supportive of FP | 4.57 (0.63, 33.21) | -- |
| Not married (legal or presumed) | 5.51 (0.72, 42.38) | 1.30 (0.69, 2.43) |
| *Reference: partner unsupportive/unsure of partner support* | *Ref.* | -- |
| **Traveled less than 30 minutes to reach health facility** | 1.25 (0.74, 2.11) | -- |
| **Number of items in Method Information Index received^c^** | 1.16 (0.92, 1.48) | -- |
| ^a^ Pills include daily combined and progestin-only oral contraceptives  ^b^ Other modern methods include condoms, fertility-awareness based methods (LAM, Two Days Method, Standard Days Method), and emergency contraceptive pills ^c^ The Method Information Index is calculated based on 3 questions: "during your visit: 1) were you informed about other methods?, 2) were you informed about side effects or problems with the method?, 3) were you told what to do if you had side effects of problems with the method?". Responses were summed to provide a count of the number of counseling items received, from 0 (received none of these counseling items) to 3 (received all items). Notes: In the full analytic cohort, 89/744 (12%) reported the most recent date that they received FP services as more than +/- 1 day of study enrollment. When we exclude these participants in this sensitivity analysis. In this sensitivity analysis, we excluded participants with a reported last FP visit greater than 1 day +/- of enrollment, for a total sample of N=449 in the derivation sample. Predictors selected using stepwise forwards and backwards to identify the Cox model with the minimum AIC value. The Efron approach was used to handle ties. The simplified model was developed by removing predictors identified in the full model that are not routinely collected (either verbally or in written documentation) in Kenyan public health facilities. | | |

**Figures**

**Figure S1. ROC curves for LASSO-Cox risk scores in the derivation and validation cohorts**

**
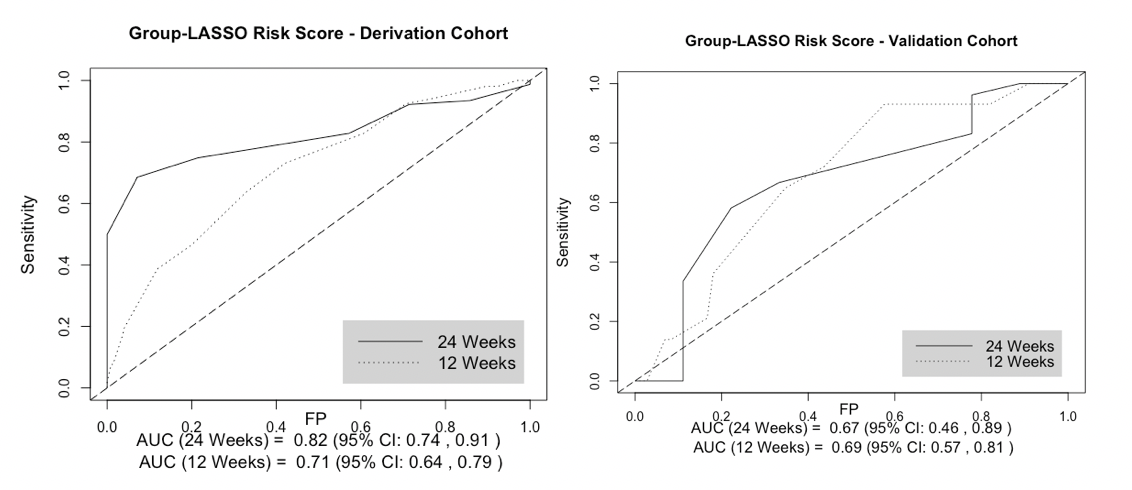
**

**Figure S2. ROC curves for full, simplified, and group LASSO risk scores in full cohort**

**
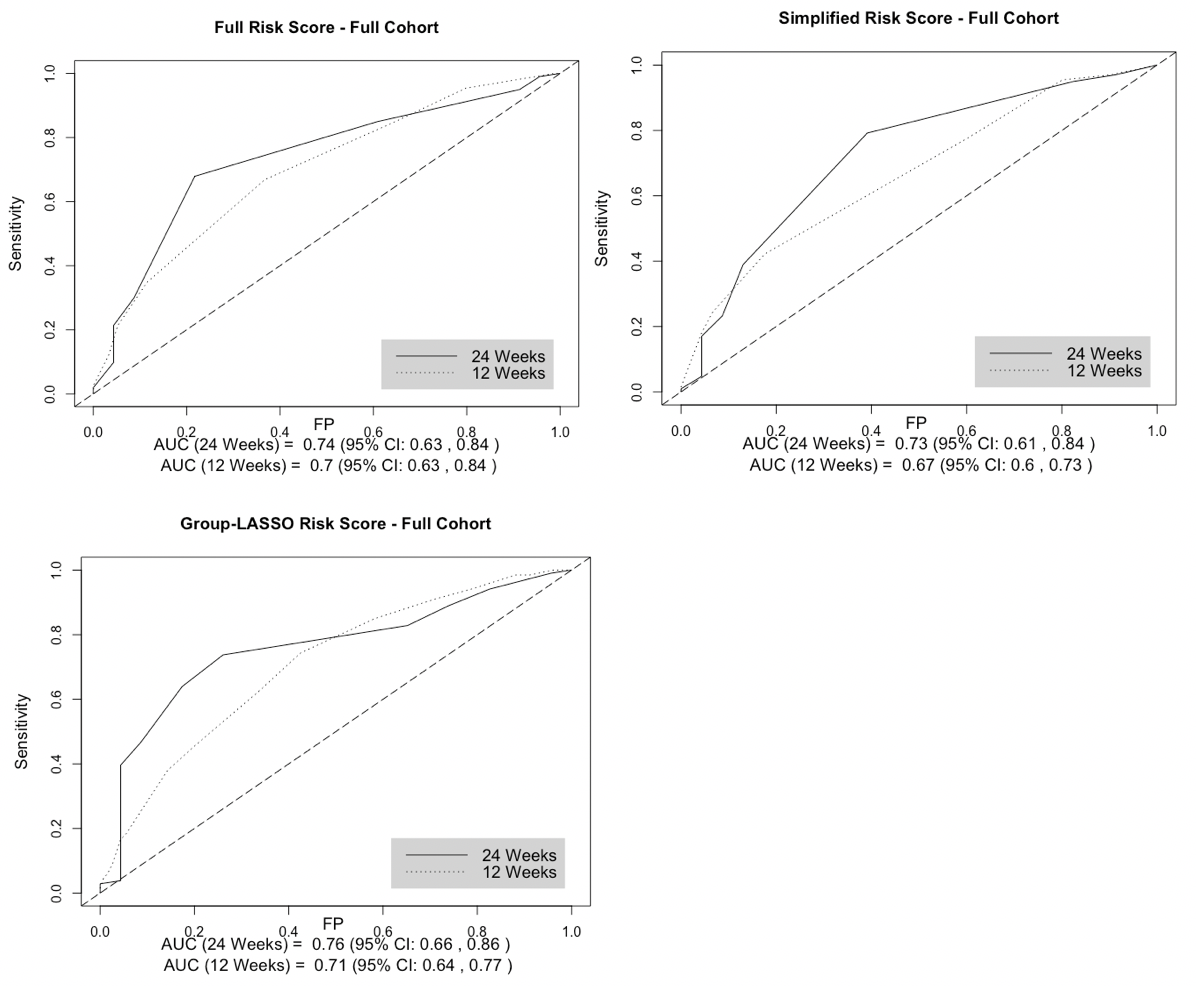
**

**Figure S3. Survival probabilities by optimal cut-point of the group-LASSO risk score**

**
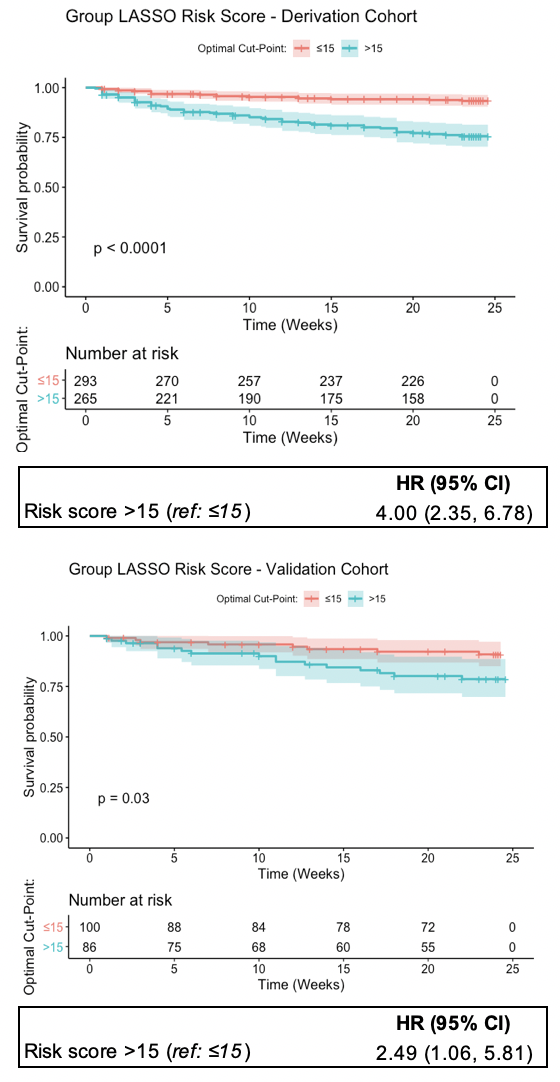
**

**Figure S4. Stated reasons for discontinuation**


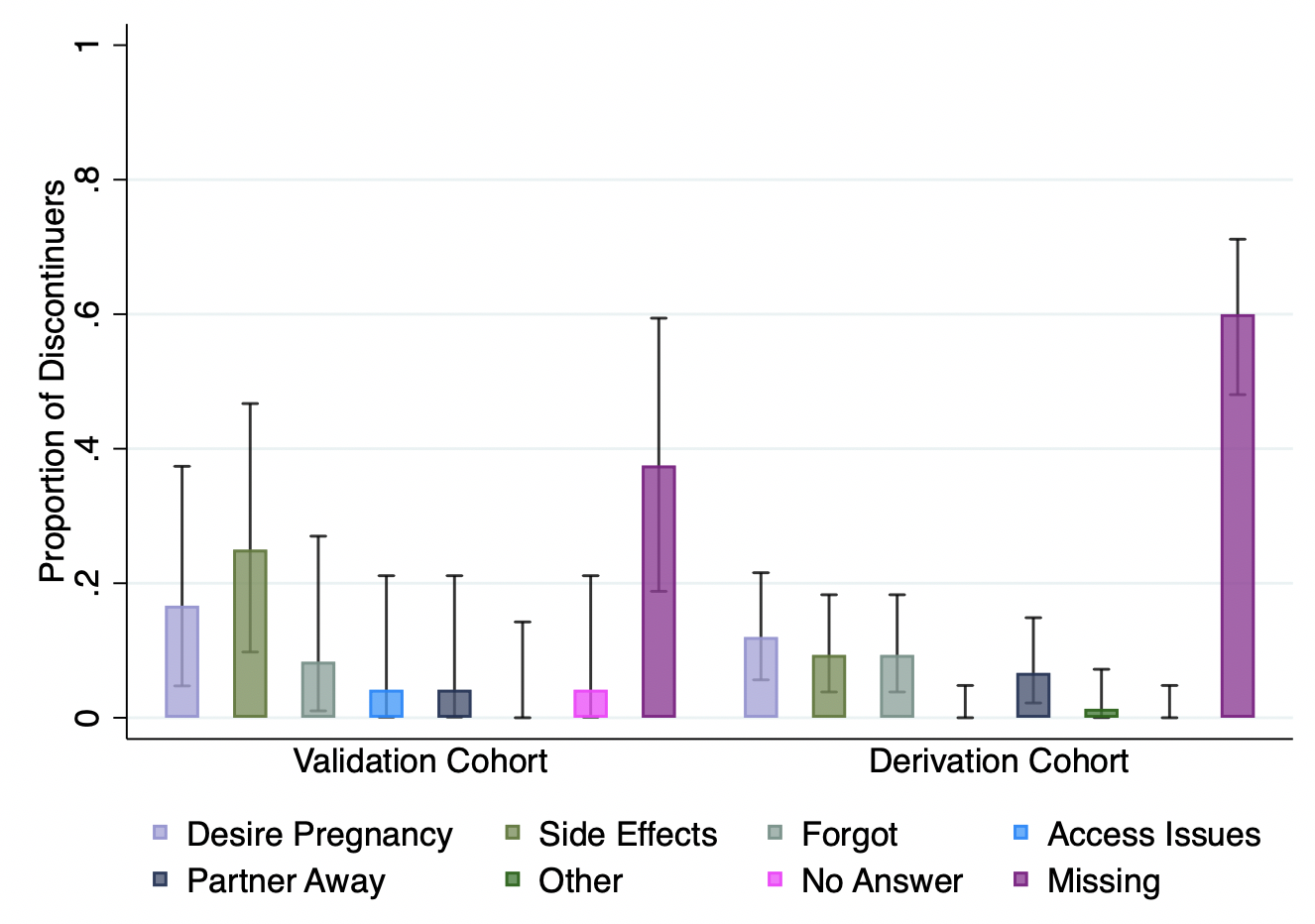

Supplement: Supplementary file 1 — Supplementary material [file mmc1.docx]
